# Melatonin as a Potent and Inducible Endogenous Antioxidant: Synthesis and Metabolism

**DOI:** 10.3390/molecules201018886

**Published:** 2015-10-16

**Authors:** Dun-Xian Tan, Lucien C. Manchester, Eduardo Esteban-Zubero, Zhou Zhou, Russel J. Reiter

**Affiliations:** Department of Cellular and Structural Biology, Health Science Center, University of Texas, San Antonio, TX 78229, USA; E-Mails: lmanchester@stmarytx.edu (L.C.M.), eezubero@gmail.com (E. E.-Z.); LunaZhou00@163.com (Z.Z.)

**Keywords:** melatonin, antioxidant, oxidative stress, synthesis, metabolism, plants

## Abstract

Melatonin is a tryptophan-derived molecule with pleiotropic activities. It is present in almost all or all organisms. Its synthetic pathway depends on the species in which it is measured. For example, the tryptophan to melatonin pathway differs in plants and animals. It is speculated that the melatonin synthetic machinery in eukaryotes was inherited from bacteria as a result of endosymbiosis. However, melatonin’s synthetic mechanisms in microorganisms are currently unknown. Melatonin metabolism is highly complex with these enzymatic processes having evolved from cytochrome C. In addition to its enzymatic degradation, melatonin is metabolized via pseudoenzymatic and free radical interactive processes. The metabolic products of these processes overlap and it is often difficult to determine which process is dominant. However, under oxidative stress, the free radical interactive pathway may be featured over the others. Because of the complexity of the melatonin degradative processes, it is expected that additional novel melatonin metabolites will be identified in future investigations. The original and primary function of melatonin in early life forms such as in unicellular organisms was as a free radical scavenger and antioxidant. During evolution, melatonin was selected as a signaling molecule to transduce the environmental photoperiodic information into an endocrine message in multicellular organisms and for other purposes as well. As an antioxidant, melatonin exhibits several unique features which differ from the classic antioxidants. These include its cascade reaction with free radicals and its capacity to be induced under moderate oxidative stress. These features make melatonin a potent endogenously-occurring antioxidant that protects organisms from catastrophic oxidative stress.

## 1. Introduction

Melatonin is a small indole molecule which was first isolated and identified in the pineal gland of cows [1]. Its originally observed function was to lighten the skin of amphibians by causing the melanin granules within the dermal melanosomes to aggregate around the nucleus of the skin cells in frogs [2]. As a result, the newly-discovered molecule was called melatonin, that is, it causes the dark pigment (melanin) in the skin to be lightened and it is also derived from serotonin. Because of this action, melatonin was once used in some patients with localized hyperpigmented skin in an attempt to reduce the pigmentation in these areas. However, this proved unsuccessful with no skin lightening effect in humans [3]. The reason is that mammalian melanosomes, unlike those in amphibians, are more or less permanently dispersed and thus, melatonin has little effect on its ability to alter pigment aggregation in the skin of mammals. 

One of the most unique features of melatonin is its circadian rhythm in vertebrates, with its secretory peak at night and low levels during the day. This feature makes melatonin a suitable signaling molecule to inform internal organs about the environmental photoperiodic alterations. Since the melatonin peak in the blood of vertebrates always coincides with the dark phase of light/dark cycle, melatonin is, thus, referred as the chemical expression of darkness [4]. In vertebrates, the normal melatonin circadian rhythm is generated exclusively by melatonin released from pineal gland since pinealectomy either eliminates this rhythm in the blood [5] or very significantly reduces its amplitude [6]. 

The pineal gland and superchiasmatic nucleus (SCN), the master clock, interact with each other; these constitute the key elements of the bio-clock which synchronize physiological activities of organisms, particularly in vertebrates, with the prevailing light/dark cycle [7,8]. SCN relays photoperiodic information to the pineal gland via sympathetic nervous system and, based on the information provided by the SCN, the pineal gland either up- or down-regulates the production of melatonin. After its release into the cerebrospinal fluid [9,10] the changing melatonin signal in turn impacts the function of the SCN. Melatonin receptors, particularly MT1, are expressed and densely populated in the SCN [11,12]. The associations among SCN, sympathetic nervous system, pineal gland and melatonin have been extensively studied and well documented [13,14,15].

The diurnal changes in melatonin levels are already apparent in primitive photosynthetic bacteria [16,17] where they are similar to those in vertebrates; however, these diurnal changes might be passive, that is, the low levels of melatonin during the photophase in bacteria might not be the result of a reduced synthesis of this indole but rather a result of depletion due to its utilization. It has been hypothesized that during the day more melatonin is metabolized in photosynthetic organisms because of its interaction with reactive oxygen species (ROS). This relates to the fact that in photosynthetic bacteria, photosynthesis is highest during the day and this process generates large quantities of ROS. Thus, the diurnal alteration of melatonin in prokaryotes may not really impact the function of their bio-clock but rather the changes are merely a coincidence of its uneven metabolism that occurs during the day and at night. This speculation is consistent with an observation of Roopin *et al.* [18]. They observed an obvious diel melatonin rhythm in a unicellular organism, a dinoflagellate of the genus *Symbiodinium.* In this species the melatonin peak occurred during dark with low levels during the day. This fluctuation disappeared, however, when this organism was exposed to constant darkness. Based on the changes in the production of oxygen resulting from photosynthesis in this organism during the light/dark cycle, the authors concluded that the oscillating pattern of melatonin in *Symbiodinium* was not driven by endogenous circadian synthesis, but rather by changes of its utilization due to the daily photocycle. Thus, the rhythm was a result of mechanisms involving the enhanced photo-consumption of melatonin by free radicals during the light hours. 

During evolution, the obvious melatonin circadian rhythm of vertebrates likely evolved from the passive alterations of melatonin production in bacteria; this rhythm then evolved to impact the bio-clock of higher organisms [19]. For example, melatonin circadian rhythm which relates to the sleep cycle is already observed in the early marine zooplankton, *Platynereis dumerilii* [20]. This organism may be the first species in which melatonin served as a signal molecule for quiescence. 

Disturbances in melatonin circadian rhythm results in chronodisruption which is associated with many health disorders including neurodegenerative diseases, heart disease, hypertension and cancer. For example, shift workers, particularly including nurses, air plane crews and miners have a higher prevalence of breast and prostate cancers [21,22,23]. A potential mechanism is that light exposure during night (their work time) suppresses their melatonin levels [24]. This is consistent with observations in animal where when tumor-bearing rats are perfused with melatonin-rich blood from a human donor (having night levels of melatonin), the growth of transplanted tumor was inhibited; however, if these rats were perfused with corresponding melatonin-deficient daytime human blood, tumor growth was promoted [25]. Thus, since light exposure during dark phase exaggerates tumor growth, light at night has been classified as a Group 2A carcinogen, *i.e.*, a probable carcinogen, by the International Agency for Cancer Research (IACR) [26]. 

As a signaling molecule, melatonin production also exhibits seasonal changes. During the winter, there is a longer nocturnal melatonin peak period due to the longer nights while in the summer the nocturnal peak is shorter [27]. This information is used by photoperiodic animals to adjust their reproductive activities to the appropriate season [28] and to determine their hibernatory behavior [29]. Without an adequate melatonin message, for example, after pinealectomy which diminishes the blood melatonin rhythm, disturbances in the reproductive capability and the hibernating cycle of photoperiodic animals are apparent [29,30,31]. 

In plants, the melatonin circadian rhythm is neither as obvious nor as consistent as in animals. High levels of melatonin during scotophase and low levels during photophase have only been observed in an evolutionarily ancient plant, *Chenopodium rubrum* [32]. Several studies have in fact shown that melatonin production in plants is stimulated by light exposure [33,34,35]. The greater the light intensity, the more melatonin is synthesized in some plants. It is known that photosynthesis generates large quantities of ROS and plants, thus, presumably produce more melatonin to protect against the toxicity of ROS [36,37]. The primary function of melatonin is as a free radical scavenger and as an antioxidant in all organisms, while the other functions of melatonin were acquired during evolution [19,38]. As a naturally occurring antioxidant, melatonin is different from the classic antioxidants in several aspects. These include the cascade reaction of melatonin with ROS as well as the fact that its synthesis is inducible in organisms under moderate oxidative stress. These unique features of melatonin make it more suitable as an endogenous antioxidant to protect organisms from stressful conditions. These features of melatonin are reviewed below.

## 2. Melatonin Synthesis

**Figure 1:**
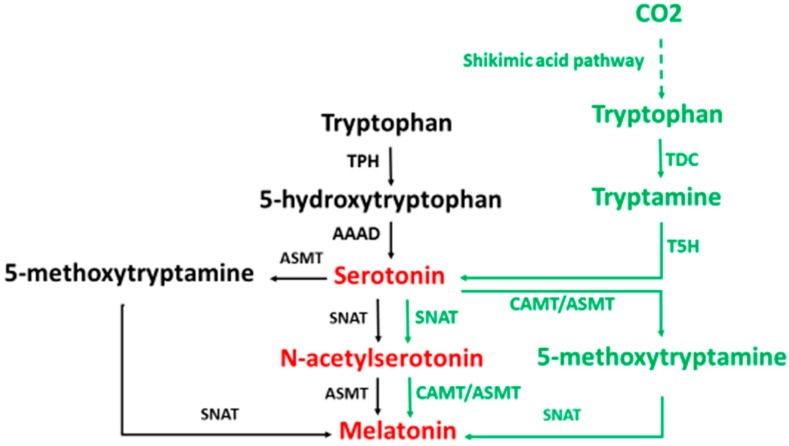
A comparison of the biosynthetic pathways of melatonin in animals and in plants. TPH: tryptophan hydroxylase; AAAD: aromatic amino acid decarboxylase; SNAT: serotonin *N*-acetyltransferase; ASMT: *N*-acetylserotonin *O*-methyltransferase, TDC: tryptophan decarboxylase; T5H: tryptamine 5-hydroxylase, CAMT: caffeic acid *O*-methyltransferase. Black occurs only in animals; green occurs only in plants; and red occurs in both animals and plants.

The majority of studies related to melatonin synthesis have been performed in vertebrates, particularly in mammals (hamster, rat, mice). In these animals, the initial precursor of melatonin synthesis is the essential amino acid, tryptophan. The classic pathway of melatonin synthesis in mammals is illustrated in Figure 1 (black and red portions).

In this pathway, serotonin *N*-acetyltransferase (SNAT) [formerly arylalkylamine *N*-acetyltransferase (AANAT)] is the most studied enzyme among those involved in melatonin synthesis. It is generally accepted that SNAT is the rate-limiting enzyme with its activity controlling the amount of melatonin produced. This idea is based on the observation that the activity of the SNAT exhibits an obvious circadian rhythm which coincides with the alterations of both pineal and circulating melatonin levels. The other enzymes involved in melatonin synthesis in animals seem to lack a circadian rhythm. During the scotophase, SNAT gene expression is significantly up-regulated in the pineal gland, likely resulting in the large rise in melatonin production [39]. However, other studies found that the increased efficiency of SNAT during night did not occur at the level of transcription but was due to the post-translational modification of this enzyme, that is, when the phosphorylation of this enzyme was modified [40,41]. For example, SNAT Thr31 phosphorylation on its own can significantly enhance melatonin synthetic efficiency (up to 7-fold) via an interaction with 14-3-3zeta which lowers the substrate K_m_; this augmented catalytic profile is largely abolished by double phosphorylation at Thr31 and Ser205 [41]. The post-translational modification mechanism controlling SNAT activity may plausibly explain the rapid rise and fall of melatonin production at the onset of dark and light, respectively. This is readily observed when pinealocyte-released melatonin is measured in dialysates collected from within the pineal gland [42]. In contrast, some researchers claim that ASMT is the rate-limiting enzyme of melatonin synthesis rather than SNAT [43,44,45]. Based on current observation, it seems that SNAT is more suitable as the rate-limiting enzyme for melatonin synthesis than ASMT since its regulatory mechanism is identified as mentioned above [40,41]. Melatonin was originally believed to be synthesized exclusively in the pineal gland. Subsequently, the retina was identified as another site of melatonin synthesis where it also exhibits a circadian rhythm [46]. Currently only one homologue of SNAT has been identified in mammals. In vertebrates other than mammals, several homologues of SNAT have been discovered. For example, at least three SNAT homologues have been identified in fish. SNAT2 is expressed exclusively in pineal gland of fish [47], and SNAT 1a and 1b are present in the retina and at other sites in this species [48]. This raises the question of origins and functions of the homologues of SNAT. Based on the nucleotide sequences of the genes, it is believed that the SNAT homologues in vertebrates have the same origin with the differences being a result of mutations during evolution. For example, the SNAT 1a and 1b are believed to be the result of whole genome duplication [48]. The various SNAT homologues may be responsible for different stimulations with enhanced melatonin synthesis. For example, some SNAT homologues may respond to photoperiodic changes and others may be up-regulated by oxidative stress. In addition, some homologues of SNAT have little to do with the melatonin production but may relate to the detoxification of toxic amines [49].

To date almost all cells, tissues and organs in vertebrates have been found to contain melatonin and are equipped with machinery for melatonin synthesis [50,51]. The largest amounts of melatonin are not produced by pineal gland but rather in the gut and skin [52,53]. It appears that the regulation of extrapineal melatonin production (except for the retina) utilizes different mechanisms from those in the pineal gland. In these organs, melatonin production is not responsive to the light/dark cycle but is regulated by the demands of the local tissues which comprise the foundation for the inducible feature of melatonin production. This will be discussed below. It has been hypothesized that mitochondria are a primary site of melatonin production [54], and these organelles do contain much higher melatonin levels than that exists in the blood. The mitochondrial melatonin levels in other tissues are independent of pineal gland, since pinealectomy failed to reduce their melatonin concentrations [55]. Mitochondrial synthesis of melatonin provides a plausible explanation as to why all cells have the capacity to produce this critical molecule. 

Melatonin synthesis in plants has a much more complicated picture than that in animals. The study of melatonin in plants has a much shorter history than that in animals. Melatonin was not identified in plants until 1995 [56,57] and its synthetic pathway was explored only recently [58]. The tentative pathway of melatonin synthesis in plants is illustrated in Figure 1 (green and red portions). There are several key differences in melatonin synthetic pathway in plants and animals: (A) Melatonin production in plants is significantly higher than that in animals; (B) Unlike animals that use the essential amino acid tryptophan, which is only available in their diet as an initial precursor of melatonin synthesis, plants can synthesize tryptophan via the shikimic acid pathway and, thus, the melatonin synthetic capacity in plants is not limited by the availability of tryptophan in their environments [38]; (C) In plants, tryptophan is first decarboxylated and then hydroxylated, while in animals this order is reversed; (D) The origins of plant SNAT and animal SNAT are completely different and they share no homology. Many homologues of SNAT as well as ASMT are present in plants; however, this is not the case in animals, especially in mammals. As a result, a truncated SNAT in some mouse strains (C57BL/6J) diminishes their melatonin production [59]. In contrast, in plants, if one homologue for melatonin biosynthetic enzyme were suppressed it would not significantly alter their melatonin production [60]; (E) The plant SNAT is much more tolerant of high temperatures than is animal SNAT. Plant SNAT functions in *N*-acetylserotonin production even at the extremely high temperature of 95 °C [58]; (F) In plants, the caffeic acid *O*-methyl-transferase (CAMT) may be the dominant enzyme in the methylation of *N*-acetylserotonin rather than the ASMT [61,62], and there is no report to show that CAMT exists in animals; (G) Chloroplasts and mitochondria both may be involved in melatonin synthesis in plants. Under normal conditions, chloroplasts are dominant sites that produce melatonin in plants. If the normal processes were blocked, for example the transcriptional suppression of tryptamine 5-hydroxylase, the dominant melatonin synthetic pathway would shift from the chloroplast to the mitochondrion [60]. 

As for the microorganisms including bacteria, yeast and unicellular microalga, they also have the capacity to synthesize melatonin at a high rate [63]. For example, during the process of wine making, large amounts of melatonin are synthesized by yeast or other bacteria [64,65,66]. In addition to melatonin, it appears that these microorganisms may also synthesize melatonin isomers [67,68,69,70,71]. In many cases, the amounts of melatonin isomers produced are significantly higher than those of melatonin. Few attempts have been made to identify melatonin synthetic pathways in microorganisms and currently little is known about the processes involved. However, yeast containing the recombinant animal melatonin synthetic enzymes including l-tryptophan hydroxylase, 5-hydroxy-l-tryptophan decarboxylase, SNAT and AMST have significantly enhanced their melatonin production [72]. It was speculated that the melatonin synthetic genes, particularly SNAT, in animals and plants were horizontally transferred from microorganisms evolutionarily [38]. Since animals and plants have completely different melatonin synthetic pathways it can be speculated that multi-origin scenarios of melatonin synthetic pathways may exist in microorganisms. Whether animals or plants inherited the microorganism’s capacity to synthesize the melatonin isomers has not been answered. To date, there is little evidence to show that animals and plants produce melatonin isomers. However, melatonin isomers derived from the microorganisms will inevitably enter the bodies of animals since billions of microorganisms are habitants of the gut of animals.

## 3. Melatonin Metabolism

**Figure 2:**
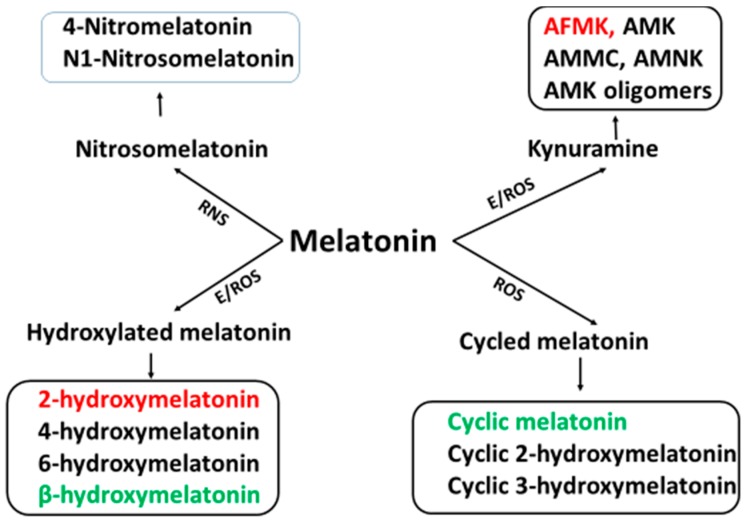
The profiles of melatonin metabolites. ROS: reactive oxygen species; RNS: reactive nitrogen species; E: enzymes including cytochrome P450 (CP450), indoleamine 2 3-dioxygenase (IDO), horseradish peroxidase (HRP), myeloperoxidase (MPO), eosinophil peroxidase (EPO), melatonin 2-hydroxylase (M2H). Black occurs only in animals; green occurs only in plants; red occurs in both animals and plants. AFMK: *N*^1^-acetyl-*N*^2^-formyl-5-methoxyknuramine; AMK: *N*-acetyl-5-methoxyknuramine; AMCC: 3-acetamidomethyl-6-methoxycinnolinone; AMNK: *N*^1^-acetyl-5methoxy-3-nitrokynuramine.

Compared to the melatonin synthesis, melatonin metabolism is less thoroughly understood. For several decades, 6-hydroxymelatonin was believed to be the only important melatonin metabolite and, thus, the majority of the investigations were focused on it. Actually, melatonin metabolism is a highly complex process and 6-hydroxymelatonin is only one of its many metabolites. Melatonin is metabolized by an enzymatic process, by a pseudoenzymatic process or via its interaction with ROS and NOS [50]. These processes are summarized Figure 2.

Melatonin is present in primitive photosynthetic bacteria including the photosynthetic prokaryote *Rhodospirillum rubrum* [16] and in cyanobacteria [73]. The former is the presumptive precursor of mitochondria, which are present in almost all cells, and the latter is the likely precursor of chloroplasts which are present in green plants. Melatonin metabolism in bacteria is an unexplored area. However, the process of melatonin metabolism in mitochondria is known. In mitochondria, melatonin is broken down via a pseudoenzymatic process. In this respect, cytochrome C functions as an enzyme to degrade melatonin to *N*^1^-acetyl-*N*^2^-formyl-5-methoxykynuramine (AFMK) [74]. Cytochrome C is a highly conservative protein that also exists in bacteria [75]. We therefore speculate that bacteria may also use cytochrome C to metabolize melatonin. Thus, cytochrome C might be the earliest protein to degrade melatonin, and AFMK might be the first product of melatonin metabolism via a pseudoenzymatic process. The catalytic center of cytochrome C for melatonin metabolism may lie in its heme molecule with a centrally-located iron atom. It has been reported that iron-containing hemoproteins such as hemoglobin interact with melatonin to form AFMK and other metabolites [76]. In this process the oxoferryl hemoprotein oxidizes melatonin and cleaves it to form AFMK [77].

During the course of evolution, other enzymatic processes became involved in melatonin metabolism. Most of the enzymes responsible for melatonin metabolism have a common feature: They are iron-containing hemoproteins similar to cytochrome C. These enzymes include cytochrome P450 (CP_450_), indoleamine 2,3-dioxygenase (IDO), horseradish peroxidase (HRP), myeloperoxidase (MPO) and eosinophil peroxidase (EPO). Each of these enzymes can cleave melatonin to form AFMK [50]. 

CP_450_ is the major enzyme for melatonin metabolism in animals. It is mainly located in the liver but is also present in other tissues. The main product of CP_450_ is 6-hydroxymelatonin and its minor product is AFMK [74]. In brain, the major enzyme for melatonin metabolism is IDO with its product being AFMK [78]. MPO and EPO are responsible for melatonin metabolism mainly at sites of inflammation. HRP is present in plants and may participate in plant melatonin metabolism. Its product, AFMK has been identified in plants [35]. Also, cyclic melatonin (different from the cyclic-3-hydroxymelatonin) and β-hydroxymelatonin have been found in plants [79]; the means of their formation are unknown. Recently, Back *et al.* [80] have found that in plants (24 different species) the dominant melatonin metabolite is 2-hydroxymelatonin and the enzyme for this reaction is designated as melatonin-2-hydroxynase [81]. Once melatonin is formed it is rapidly metabolized to 2-hydroxymelatonin. In the plants tested, the average ratio of melatonin to 2-hydroxymelatonin was 1:368. If this ratio is confirmed by others, it would indicate that the melatonin synthetic capacity in plants is even more efficient than previous expectations.

Interestingly, melatonin can also be metabolized by non-enzymatic process. Melatonin interaction with ROS and NOS generates a variety of metabolites. These include cyclic 3-hydroxymelatonin, 2-hydroxymelatonin, 4-hydroxymelatonin, *N*-nitrosomelatonin, *N*-(1-formyl-5-methoxy-3-oxo-2,3-dihydro-1*H*-indol-2-ylidenemethyl) acetamide, AFMK, AMK *etc.* The metabolites of enzymatic events, pseudoenzymatic processes, and ROS interaction pathway are common to each other. Thus, it is difficult to determine which metabolic process is dominant under *in vivo* conditions. During severe oxidative stress, however, it is assumed that the majority of melatonin would be metabolized by ROS interactions since oxidative stressors significantly reduce the total organismal melatonin levels (see below). The complexity of melatonin metabolism poses a challenge for researchers. To date, little is known regarding melatonin metabolism in plants and virtually nothing is known about melatonin metabolism in microorganisms. We believe that additional pathways of melatonin metabolism and many novel melatonin metabolites will be uncovered in the coming years.

## 4. Cascade Reactions of Melatonin: Interactions with ROS and NOS

**Figure 3:**
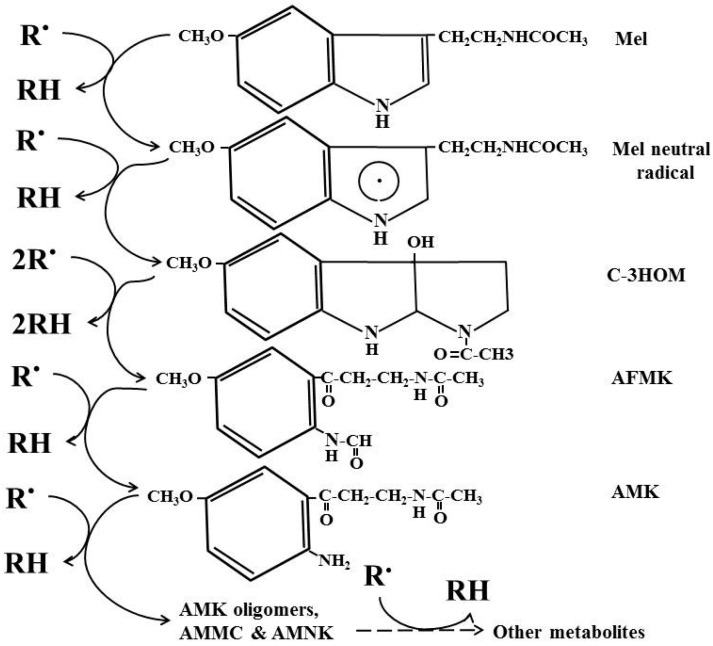
Cascade reaction of melatonin interaction with free radicals and its mebabolites. R.: radical; RH: reduced agent; Mel Melatonin; C-3HOM: cyclic 3-hydroxymelatonin; AFMK: *N*^1^-acetyl-*N*^2^-formyl-5-methoxyknuramine; AMK: *N*-acetyl-5-methoxyknuramine; AMCC: 3-acetamidomethyl-6-methoxycinnolinone; AMNK: *N*^1^-acetyl-5-methoxy-3-nitrokynuramine; dashed arrow: unidentified reactions.

As a potent free radical scavenger and antioxidant, the cascade reaction of melatonin distinguishes it from other classic antioxidants. That melatonin and its secondary and tertiary metabolites are able to neutralize numerous toxic oxygen derivatives is referred as its cascade. Via this means, one melatonin molecule has the capacity to scavenge up to 10 ROS *vs.* the classic antioxidants that scavenge one or less ROS. The antioxidant capacity of melatonin has been compared with other antioxidants including vitamin C, vitamin E, glutathione and NADH in both *in vitro* and *in vivo* conditions [82,83,84]. In most cases, melatonin is superior to these molecules. Of particular note is the ability of melatonin to protect cells against oxidative stress more efficiently than other antioxidants under *in vivo* conditions [85,86,87,88]. An important reason for this is its cascade reaction with ROS and NOS. Thus, the products (or metabolites) of melatonin following its interaction with ROS and NOS retain the ability to scavenge free radicals (Figure 3). In this respect, one melatonin molecule has a capacity to detoxify numerous toxic ROS or NOS. In contrast, the scavenging ratio of other antioxidants with ROS or NOS is 1:1 or less.

Several studies have reported that the melatonin metabolite, cyclic-3-hydroxymelatonin, is more potent than melatonin to scavenge the hydroxyl radical and other ROS [77,89,90,91]. This is also the case for its tertiary metabolite, AMK [92,93,94]. AFMK also is a central molecule of melatonin metabolism both during its enzymatic break down and during its interaction with ROS or NOS. A cyclic voltammetry study has shown that AFMK exhibits two anodic waves at Ep(a) of 456 and 668 mV, respectively. This property indicates that AFMK donates two electrons at different potentials to function as a reductive molecule. As a result, AFMK can donate an additional electron to neutralize radicals in contrast to the classic small molecular antioxidants including vitamin C and E [95].

A recent theoretical study suggested that AFMK is a balanced antioxidant compared to other antioxidants tested. Based on its electron donation and accepting capacity, it positions itself in the middle of the tested antioxidants [96]. In *in vitro* studies, the antioxidant capacity of AFMK is not as strong as its precursor melatonin [94,97]; however, AFMK and its metabolite AMK also exhibit strong anti-inflammatory actions by inhibiting prostaglandin formation [93,98]. Indeed, the anti-inflammatory activity of melatonin [99] may in major part be mediated by AFMK. In *in vivo* studies, AFMK exhibits strong protection against oxidative stress [100,101]. The chain reaction of melatonin and its metabolites with the ROS or NOS has come to be known as the free radical scavenging cascade [102]. This cascade reaction not only amplifies the efficiency of melatonin as an antioxidant but also expands its scavenging spectrum. Thus, melatonin can scavenge a variety of ROS and NOS including hydroxyl radical, H_2_O_2_, O_2_**^−^**, singlet oxygen, NO, NOO**^−^**, hypochlorite radical, LOO**^−^** *etc.* This cascade of reaction with ROS and NOS contributes to its superior protective effects in animal and human studies against oxidative stress [103,104]; this also expands melatonin’s use in industrial fields. Based on this antioxidant cascade, scientists have successfully used melatonin to replace the classic reducing agent, hydrozine used in generating graphene, a novel industrial material that functions as a semiconductor and as a bacterial wrap. Melatonin converts graphene oxide to graphene and stabilizes the structure of graphene much longer than the classic reducing agent [105,106]. This is probably attributable to melatonin and its secondary and tertiary metabolites which sequentially scavenge ROS in the reduced graphene.

## 5. Melatonin, an Inducible Antioxidant

Different from most other antioxidants, which are exclusively obtained from the diet, melatonin is both derived from diet and is also generated endogenously. For the exclusive diet-derived antioxidants a deficiency is more likely to develop when the diet is inadequate. For melatonin, it seems more resistant to an insufficient supply from the food since it can be synthesized by virtually all organisms. There is no report to date that documents a melatonin deficiency due to a poor diet. In most cases, melatonin deficiency is the result of its reduced production or its elevated consumption. For the former, aging is the common cause. In most organisms melatonin production is jeopardized in advanced aging [107,108,109,110]. A mutation of the gene that determines melatonin synthetic enzyme activity is another cause. For example, mice with the truncated SNAT are reported to be melatonin deficient compared to other mouse stains [59,111]. Melatonin also can be exhausted by severe oxidative stress where melatonin degradation by free radicals is presumably much faster than its production [112,113]. Likewise, in oxidative stress-related disorders such as in the Alzheimer’s disease and cardiovascular disease, melatonin levels are lower than those of the healthy population [114,115,116,117,118,119]. In other cases, a melatonin deficiency can be a result of external or environmental toxicity. Rats treated with a strong oxidant such as 2,3,7,8-tetrachlorodibenzo-*p*-dioxin (TCDD) also experience reduced melatonin levels [120,121,122]. Certainly, the dual sources of melatonin in organisms make it much more reliable as an antioxidant than others when it is needed. 

In addition to its antioxidant scavenging cascade, another important characteristic of melatonin as a free radical scavenger and antioxidant is its deducibility by moderate oxidative stress or unfavorable environmental conditions. This was observed decades ago. For example, forced swimming or cold stressing rats increased their melatonin production via an elevated SNAT activity to match the external stressors [123,124,125,126]. In food-restricted rats high levels of melatonin also were detected [127]; this was also found in a non-human primate, the monkeys [128]. Food restriction is considered a minor stressor and it significantly prolongs the life span of rodents [129,130]. Increased melatonin production would likely be a beneficial factor since many studies have proven that melatonin exhibits longevity-promoting effects in different organisms from yeast, rotifer, *Drosophila* and mammals [19,110].

This phenomenon is more obvious in hibernating animals. During hibernation, the brain blood supply drops to less than 10% compared to euthermic animals; this is considered a type of brain ischemia. Upon arouse, the brain blood supply rapidly returns to normal. This process is referred as a physiological ischemia/reperfusion [131]. Theoretically, this physiological ischemia/reperfusion would normally very likely injure the neurons; however, the neuronal injury does not occur in hibernating animals. No injury occurs because when the brain is experiencing reperfusion melatonin levels are enhanced significantly, thus protecting the brain from ischemia/reperfusion injury [131]. This protection may be a consequence of melatonin’s inducibility under cold stress. In yet another study, when caerulein, a chemical that causes pancreatic oxidative stress was injected into rats, SNAT gene expression in the pancreas was up-regulated by 2.5 fold [132]. Since in mammals, no isoform of the SNAT has been identified, it appears that oxidative stress up-regulates the sole mammalian SNAT to enhance melatonin production; however, the mechanism by which oxidative stress mediates this response remains unknown.

The phenomenon of stress-induced melatonin production has been extensively studied in plants. As predicted, plants must often survive in unfavorable environmental conditions including extreme cold, heat, strong solar irradiation, and soil pollution due to heavy metals and chemicals; each of these has been found to significantly up-regulate melatonin production in plants so they can cope with toxic environmental stressors [133,134,135,136]. In contrast to animals, many homologues of plant SNAT and ASMT have been identified [137,138,139]. It seems likely that each isoform of these enzymes exhibits a different response to the variable environmental stressors regarding the enhancement of melatonin production. For example, in apple different isoforms of SNAT are responsible for melatonin synthesis during different stages of apple maturation [137]. 

The inducible feature of melatonin by oxidative stress or unfavorable environments in organisms already exists in unicellular organisms. When microalgae *Gonyaulax polyedra* were exposed to low temperature, they significantly up-regulated their melatonin production to increase their tolerance to the cold temperature [140]. Similarly, Tal *et al.* [134] systemically investigated the responses of a macroalga (*Ulva* sp.) to oxidative stress in terms of their melatonin production. They found that elevated ambient temperature or treatment of these organisms with heavy metals significantly stimulated their melatonin levels and protected them from oxidative damage. To induce the protective mechanisms under moderate stress is an important survival strategy for organisms to prepare them to cope with the subsequent and often more severe stress. Based on the evidence, it appears that very early in evolution, up-regulation of melatonin production was selected as a survival strategy. The inducible feature of melatonin under stressful conditions has been inherited by perhaps all organisms including plants and animals. The inducibility of melatonin under stressful conditions has manifested it as a unique antioxidant to effectively protect organisms from catastrophic oxidative stress. 

## 6. Concluding Remarks

Due to its obvious circadian rhythm, melatonin was selected by multicellular organisms to serve as a signaling molecule with this message allowing for the photoperiod to synchronize physiological events such as seasonal reproduction and hibernation. From an evolutionary point view, however, the original and primary function of melatonin serves as the free radical scavenger and antioxidant; this is consistent with the existence of melatonin in primitive photosynthetic bacteria. During evolution, the genes for melatonin synthesis of these bacteria were horizontally transferred to other species. As seen in Figure 1, the melatonin synthetic pathways are significantly different in animals and plants. This indicates multiple origins of the melatonin synthetic genes. Currently, little is known as to how melatonin is synthesized in microorganisms and which genes are involved. The lack of information hinders our understanding as to the origins of melatonin synthetic genes in animals and plants. 

Compared to melatonin synthesis, melatonin metabolism is even more complicated. We point out that the iron-containing hemoprotein is a key structure for melatonin metabolism. Thus, we hypothesize that cytochrome C might be the original protein (or enzyme) which was responsible for melatonin metabolism in bacteria. All other enzymes for melatonin metabolism presumably evolved from cytochrome C. These include CP_450_, IDO, HRP, MPO, EPO and probably others. Melatonin is metabolized via the enzymatic, pseudoenzymatic or free radical interactive processes. The resulting products are common to these processes, so it is difficult to identify which process is dominant. However, under excessive oxidative stress the free radical interaction is expected to be the dominant pathway for melatonin metabolism. As a result of multiple metabolic pathways, it seems likely that new melatonin metabolites will be identified. 

As an antioxidant, melatonin has several unique features differing from those of classic antioxidants. These include the cascade pathway for scavenging numerous free radicals and its inducible capacity under stressful conditions. That melatonin, due to its secondary and tertiary metabolites, is able to neutralize numerous toxic oxygen derivatives is referred as its cascade. Via this means, one melatonin molecule has the capacity to scavenge up to 10 ROS *vs.* the classic antioxidants that scavenge one or less ROS. The cascade reaction of melatonin’s interaction with ROS amplifies its ability as a potent antioxidant. This also explains why under *in vivo* condition, melatonin typically performs better than a classic antioxidant against oxidative stressors. In addition to its cascade reaction, the production of melatonin in organisms is inducible under conditions that promote oxidative stress. The inducible feature of melatonin appeared at an early stage of evolution since unicellular organisms, particularly, algae, already possess this capacity. The stress-induced capacity of melatonin production can precondition organisms to cope with potential catastrophic oxidative stresses and improve their survival chances under extremely harmful environmental conditions. 
